# Mechanical Properties and Energy Evolution of Fractured Sandstone under Cyclic Loading

**DOI:** 10.3390/ma15176116

**Published:** 2022-09-02

**Authors:** Xinwei Li, Zhishu Yao, Xianwen Huang, Xiaohu Liu, Yu Fang, Yongjie Xu

**Affiliations:** 1 School of Civil Engineering and Architecture, Anhui University of Science and Technology, Huainan 232001, China; 2 School of Civil Engineering, Suzhou University of Science and Technology, Suzhou 215009, China

**Keywords:** fractured sandstone, deformation modulus, lateral expansion coefficient, viscoelasticity, energy evolution

## Abstract

Affected by fracture distribution, sandstone shows different deformation and energy evolution characteristics under cyclic loading and unloading conditions. Therefore, uniaxial cyclic loading tests were conducted on fractured sandstone with different angles. The deformation characteristics and the evolution law of energy indexes with the peak load and crack angles were obtained under cyclic loading. Studies have shown that: The deformation modulus of sandstone first increases and then decreases, and the lateral expansion coefficient is positively correlated with the peak load. Based on the viscoelastic deformation theory, an energy analysis model considering damping energy and damage energy is established. The dissipated energy can be divided into the damping energy consumed to overcome rock viscoelasticity and damage energy causing damage by viscoelastic deformation theory. Based on this model, the relationship between elastic property, damping energy, damage energy and fracture angle is obtained, and the damage energy increases slowly first and then rapidly. The research results provide a reference for predicting the damage and failure of rock.

## 1. Introduction

In underground engineering, there are various levels of defects within the rock mass, such as faults, cracks, cracks, holes, voids, gaps, pores, etc. These defects exist in rock bodies in different spatial scales and forms; under the disturbance stress of blasting and excavation, the rock is often in the state of cyclic loading and unloading, which often control the deformation, destruction and stability of the corresponding rock mass engineering [1,2,3,4,5,6]. Thus, it is particularly important to study the deformation and failure mechanism of fractured rock under cyclic loading.

To date, some researchers have conducted substantial basic research on fractured sandstone. Tian et al. [7] studied double V-shaped prefabricated fissures; research shows that V-shaped flaw properties slightly influence the crack initiation positions but significantly influence the crack trajectories. Xu et al. [8] conducted a cyclic loading test on cracked sandstone and found the relationship between strength and crack angle. Yin et al. [9] conducted shear tests on fractured marl and studied the mechanical properties and failure modes. Their research shows the failure on both sides is generally a tensile-shear mixed failure, in which the tensile failure is mainly concentrated in the middle of both sides. Zhang et al. [10] conducted experiments on preflawed sandstone to investigate the infrared radiation characteristics during the failure process and found the precursor point for preflawed rock failure is identified based on the CVIRT-time curve, with an average precursor point of 83% of the peak stress. Ghamgosar et al. [11] carried out the rock fracture process test under cyclic loading. Wang et al. [12,13] conducted increasing amplitude fatigue loading experiments with different stress amplitudes to investigate the fracture of granite samples. Their research shows that rock volumetric deformation increases and damage propagation accelerates with increasing fatigue loading stages. Zhong et al. [14] studied the fracture properties of jointed rock infilled with mortar under uniaxial compression. Sun et al. [15] conducted impact failure tests on sandstones with different fissure numbers and fissure dips. They simultaneously recorded the crack growth after each impact and found that as fissure dip increases and the number of fissures increases, the dynamic peak stress and dynamic elastic modulus of the fractured sandstone gradually decrease. Suner et al. [16] studied the influence of natural fractures on the mechanical response and failure mechanisms of stone mine pillars. Erarslan et al. [17] investigated the effect of cyclic loading on fracture propagation, which testifies that the opened fracture tip can be completely closed during the unloading period. The above authors have carried out many experiments on fractured rocks, studied the effects of different loading modes and forms of fractures on the mechanical properties and fracture morphology of sandstone, and achieved fruitful results. However, most existing studies describe the destructive phenomena of fractured rocks, and there are few studies on the energy evolution and failure nature of fractured rocks.

The combination of high-speed photography and digital image correlation (DIC) technology is the most promising and ideal means to observe crack growth and analyze the failure mechanism of rock-like materials [18]. Mansour et al. [19] couple the 3D printing technology with the digital image correlation (DIC) and the bonded particle model (BPM) to study the failure of rock-like specimens with pre-existing flaws. Wei et al. [20] carried out a uniaxial compression experiment of sandstone specimens, and the DIC system captured the rock’s whole field deformation and failure characteristics. Wang et al. [21] used DIC technology to analyze the failure mechanism of rock. They found that the fine-grained sandstone undergoes five critical moments under impact load: initiation of the first crack, initiation of the second crack, crack penetration, local crushing, and thorough breaking.

Rock deformation and instability under stress are inevitably accompanied by energy transformation; its evolution process can reflect the failure process of rocks. Research on rock failure mechanisms is mostly based on thermodynamic theory. Rock deformation and failure is an energy-driven instability failure, a process of energy absorption and release [22,23,24,25,26]. Peng et al. [27] conducted a uniaxial cyclic loading and unloading test under stress gradients with a constant lower load limit on sandstone containing cracks at different angles. They quantified the relationships of crack angles with the energy storage coefficient and the energy dissipation coefficient. Kim et al. [28] studied estimating the damage evolution of in-situ rock mass by acoustic emission technology under increasing cyclic loading. Tan et al. [29] concluded that energy dissipation shows good consistency with the damage evolution. Meng. et al. [30] studied the acoustic emission characteristics and energy evolution characteristics of rock. They concluded that rock’s internal structure and axial loading stress were the main factors affecting energy storage. Based on the principle of energy evolution and the hysteresis effect of concrete, Pei et al. [31] conducted triaxial cyclic loading and unloading tests to study the influence of confining pressure on the energy storage limit, energy storage ratio, and energy consumption ratio. Through analysis of the energy evolution characteristics of the hysteresis loop of the stress-strain curve. Li et al. [32] established a damage model for fractured rock from the perspective of energy dissipation, which considered the initial damage and the concept of equivalent modulus. Liu et al. [33] conducted a triaxial test on four different strength types of rock samples, and the relationship between energy evolution and crack growth was obtained.

These scholars have researched the mechanical properties, failure modes, and energy evolution of the rock deformation and failure process, providing an important reference for this study. However, scholars did not consider the energy dissipated by rock viscoelasticity under cyclic loading and unloading [34,35], so the influence of fracture angle on energy evolution is not considered. Thus, this study considers sandstone as the research object and conducts cyclic loading tests to analyze the mechanical response of fractured sandstone with different angles under cyclic loading. Given the viscoelastic effect, fractured sandstone’s energy evolution law is analyzed from different angles.

This paper simulated the fractured rock by prefabricated cracks and simulated the disturbance stress by graded cyclic loading and unloading. Unlike previous research, in this study, the influence of stress history on the mechanical properties of fractured sandstone is eliminated, and each cyclic’s deformation characteristics are analyzed. The study of the energy index in each stage of deformation and failure in fractured sandstone is emphasized. In the energy analysis, the damping energy that overcomes the viscoelasticity consumption of fractured sandstone is distinguished from the damage energy that causes damage, which is more consistent with actual conditions. The research results provide a theoretical basis for understanding fractured sandstone’s energy conversion mechanism and deformation under cyclic loading.

## 2. Experimental Study

### 2.1. Test Preparation

The sandstone specimens used in this test were taken from a mine in Huainan (Anhui, China). All specimens were from the same rock block without obvious cracks or other defects.

The sandstone was formed into a 50 mm ×100 mm standard cylindrical test block using a coring drilling machine and a grinding machine; the parallelism of the two end faces was greater than 98%. Holes with a diameter of 2 mm were drilled in the middle of the specimen using a drill rig. A diamond wire was passed through the drilling hole; specimens with a crack length of 16 mm and dip angles of 0°, 30°, 45°, 60°, and 90° were processed. The wave velocity was measured with an MC-6310 non-metallic ultrasonic detector to eliminate the effects of anisotropy and discreteness; the wave velocity of the specimens was approximately 2.4 km/s.

### 2.2. Test Scheme and Results

**Figure 1:**
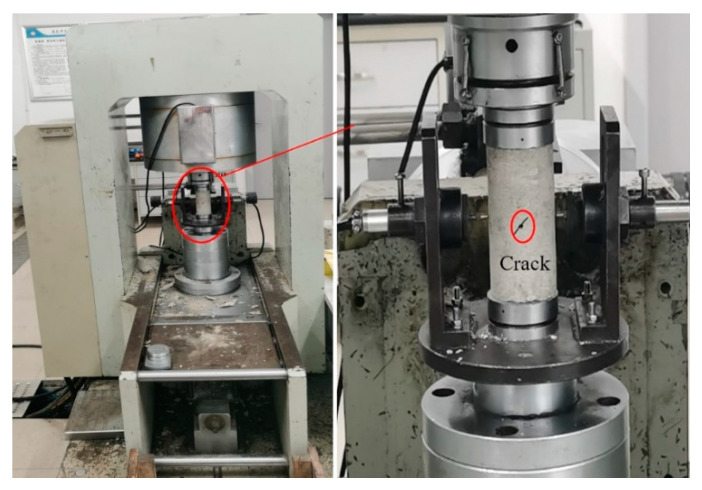
RMT–150B electro-hydraulic servo-controlled test machine system.

As shown in Figure 1, the RMT-150B electro-hydraulic servo-controlled test machine system, which can realize the load or displacement control, was used as the load system to characterize the sandstone mechanical behaviors.

**Figure 2:**
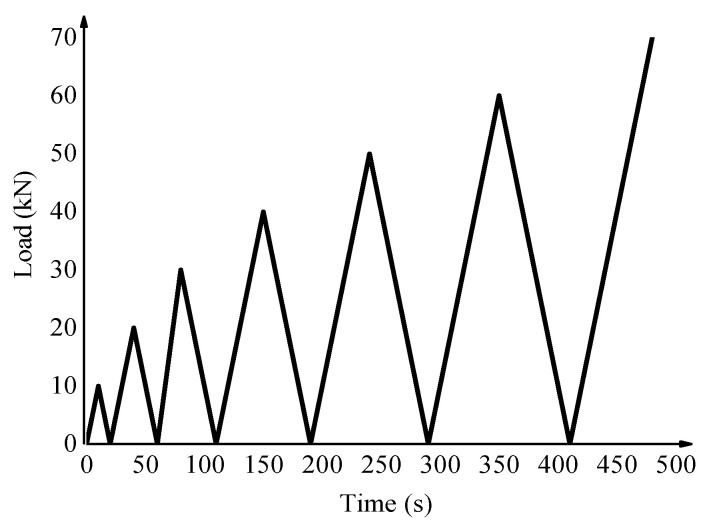
Stress path of cyclic loading test.

Loading control mode was used in the cyclic loading test; the loading rate was 1 kN/s. To obtain enough cycles and minimize the impact of stress history, the peak load in the first cycle of test pieces was set as 10 kN, and the load increased by 10 kN for each cycle. As shown in Figure 2, the loading mode was 0 kN–10 kN–0 kN–20 kN–0 kN–30 kN, etc., until the specimen was destroyed.

## 3. Analysis of Mechanical Properties of Fractured Sandstone under Cyclic Loading

### 3.1. Characteristics of Fractured Sandstone Stress-Strain Curve under Cyclic Loading

**Figure 3:**
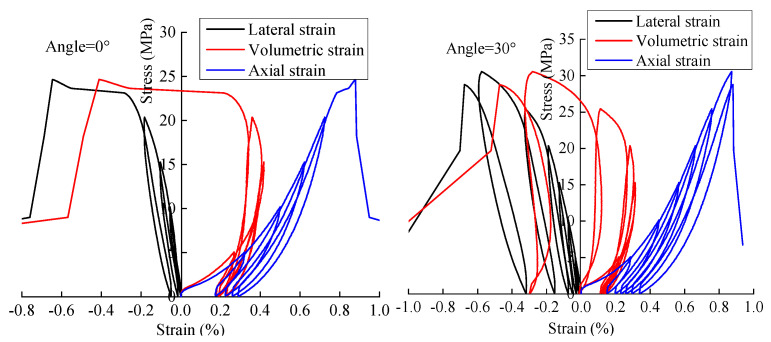

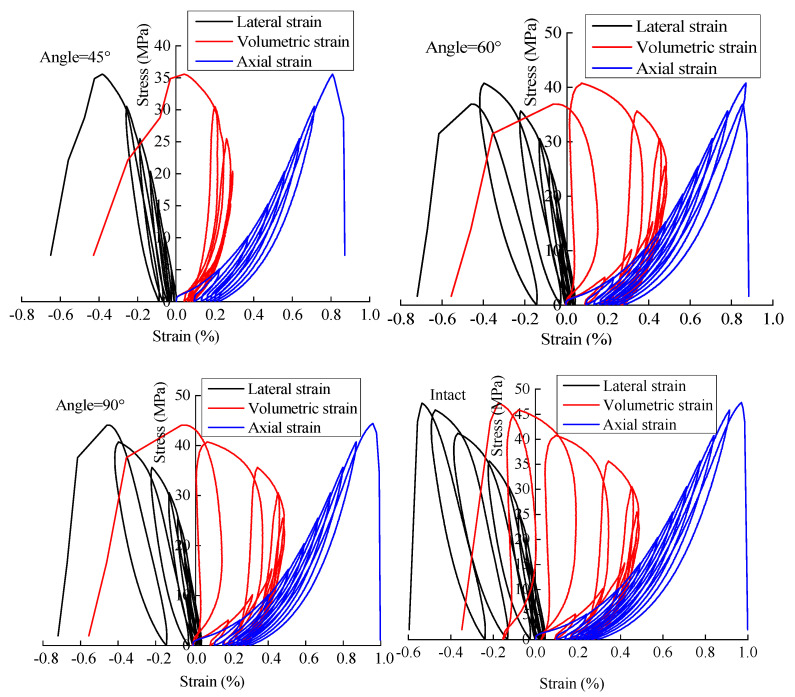
Stress-strain curves of specimens under cyclic loading.

Figure 3 shows typical stress-strain curves of specimens. As shown in Figure 3, each cycle generated residual strain; the lateral stress-strain curve gradually moves to the left, and the axial stress-strain curve gradually moves to the right. The stress-strain curve of rock noticeably deviates from the historical stress-strain curve before failure. When the stress level is low, the development trend of the volume stress-strain curve is the same as that of the axial stress-strain curve. When the stress exceeds approximately 0.5 $\sigma_{c}$, the cracks parallel to the principal axis expand greatly under the load. The development trend of the volume stress–strain curve is the same as that of the lateral strain, indicating that when the stress is low, the volume strain is controlled by the axial strain. When the stress reaches a certain level, the lateral strain controls the volume strain.

Table 1 shows the sample number and basic parameters. The horizontal projection area is the projection area of the prefabricated crack in the horizontal direction. The horizontal projection area of the crack surface + the effective bearing area = the horizontal projection area of the specimen. The peak strength is the failure strength of the specimen under cyclic loading.

### 3.2. Variation of Deformation Modulus and Expansion Coefficient

There are many ways to determine the elastic modulus, including E_50_, E_t_, the slope of the approximate straight line portion of the elastic segment, and the secant modulus of the unloading curve. The value method of Poisson’s ratio is similar. Sandstone is a viscoelastic plasticity material; it is not sufficient to use the elastic modulus E and Poisson’s ratio μ to characterize the deformation properties of sandstone. To characterize the deformation characteristics of each cycle and eliminate the influence of stress history, the secant slope of the loading section of each cyclic stress-axial strain curve was used as the deformation modulus. The secant modulus of the loading section of each cycle stress–lateral strain curve was used as the lateral expansion coefficient, and the deformation characteristics of sandstone under cyclic loading were comprehensively analyzed.

(1) $$E_{i}^{}=\frac{\sigma_{i\max}^{1}-\sigma_{i\min}^{1}}{\varepsilon_{i\max}^{1}-\varepsilon_{i\min}^{1}}$$

(2) $$\mu_{i}^{}=\frac{\varepsilon_{i\max}^{3}-\varepsilon_{i\min}^{3}}{\varepsilon_{i\max}^{1}-\varepsilon_{i\min}^{1}}$$

where $E_{i},\mu_{i}$ are the deformation modulus and the lateral expansion coefficient, respectively; $\sigma_{i\max}^{1},\sigma_{i\min}^{1},\varepsilon_{i\max}^{1},\varepsilon_{i\min}^{1},\varepsilon_{i\max}^{3},\varepsilon_{i\min}^{3}$ are the *i*th peak stress, initial stress, peak axial strain, initial axial strain, peak lateral strain, and initial lateral strain, respectively. The formula reflects the deformation characteristics of a single cycle. It eliminates the influence of accumulated residual strain caused by the stress history on calculating the deformation modulus and the lateral expansion coefficient.

**Figure 4:**
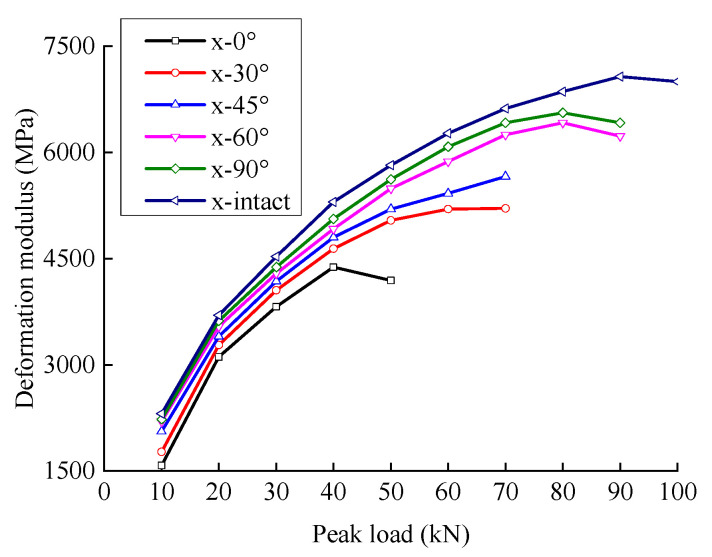
Relationship between elastic modulus and load level.

As shown in Figure 4, the deformation modulus increases and decreases with the peak load because the cyclic loading compacts the rock, increasing the stiffness and the deformation modulus. However, when the load increased to a certain level, the micro-crack pores in the rock expanded through the ability to resist deformation was weakened, and the modulus of deformation decreased.

**Figure 5:**
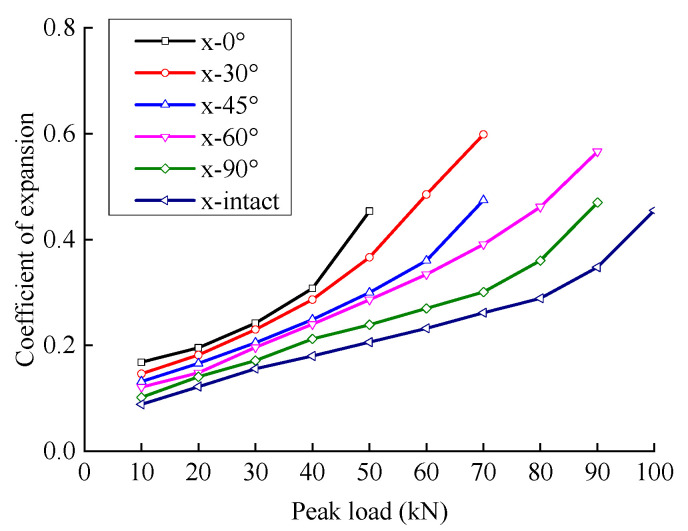
Relationship between the coefficient of expansion and load level.

As shown in Figure 5, with an increase in peak load, the lateral expansion coefficient of rock first increases slowly. It then increases rapidly because a greater load generates a greater tensile stress from the Poisson effect. Faster crack propagation and connection in the direction of the parallel principal axis, resulting in faster growth of the transverse strain and the lateral expansion coefficient.

When the crack angle was less than 45°, there was a large gap between the fractured sandstone’s deformation modulus and lateral expansion coefficient and the intact specimen. When the crack angle was greater than 60°, the difference between the deformation modulus and the lateral expansion coefficient of the fractured sandstone was small compared to the intact specimen. This indicated that the influence of the crack angle on the deformation resistance of sandstone is nonlinear and that a smaller crack angle produces a greater decrease in the deformation resistance.

The deformation modulus increased with an increase in the crack angle; the deformation modulus of the intact specimen was the largest. The lateral expansion coefficient decreased with an increase in the crack angle; the lateral expansion coefficient of the intact specimen was the smallest. The analysis shows that a larger crack angle produces a smaller horizontal projection of the crack surface and a larger effective bearing area. The deformation resistance of the fractured sandstone is closer to that of the complete specimen.

## 4. Analysis of Energy Evolution

### 4.1. Energy Conversion Theory under Cyclic Loading

Rock failure is essentially an energy-driven instability phenomenon. It is assumed that there is no heat exchange in the rock deformation and failure process, and acoustic emission and radiant energy are ignored. According to the energy conservation law, the work done by an external force is the total input energy. The relationship between input energy, elastic energy, and dissipated energy can be expressed as [36,37,38,39].

(3) $$U_{i}=U_{ei}+U_{di}$$

(4) $$U_{i}=\int_{\varepsilon_{O}}^{\varepsilon_{A}}\sigma_{i}^{+}d\varepsilon_{i}$$

(5) $$U_{ei}=\int_{\varepsilon_{C}}^{\varepsilon_{A}}\sigma_{i}^{-}d\varepsilon_{i}$$

(6) $$U_{di}=U_{i}-U_{ei}=\int_{\varepsilon_{O}}^{\varepsilon_{A}}\left(\sigma_{i}^{+}-\sigma_{i}^{-}\right)d\varepsilon_{i}$$

where $U_{i},U_{ei},U_{di}$ represent input energy, elastic energy, and dissipated energy in the *i*th cycle, respectively; $\sigma_{i}^{+},\sigma_{i}^{-}$ represent the loading curve and unloading curve of the *i*th cycle, respectively.

### 4.2. Energy Conversion Theory Considering Viscoelastic Effect

Rock is not a continuous, isotropic, and homogeneous ideal material, and the friction between fracture surfaces and the viscosity between liquids produce a nonlinear hysteresis effect during the loading process. In a previous analysis of energy evolution under cyclic loading, the area enclosed by the cyclic loading and unloading curves at each stage was generally regarded as the dissipated energy causing damage, residual deformation, and overcoming damping work. The energy dissipated to cause plastic damage and overcome viscosity is not specifically divided. Thus, based on viscoelastic deformation theory, the dissipated energy is subdivided into plastic damage energy and damping energy. The damage energy (plastic damage energy) is defined as the energy that causes the initiation and expansion of microcracks, the plastic deformation of rock, and the deterioration of stiffness.

**Figure 6:**
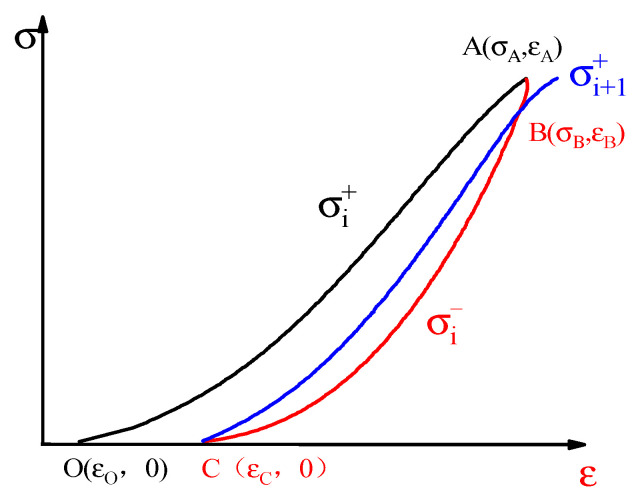
Diagram for calculation of energy.

The viscoelastic deformation characteristics of rock were considered, the dissipated energy was divided into damping and damage energy, and a new energy analysis method was established. Figure 6 shows a diagram of the energy calculation for rock under cyclic loading. The unloading curve of the previous cycle and the loading curve of the next cycle intersect at point B, the physical significance of which is that there is a point B on the unloading curve. When the stress at this point is unloaded to 0 and then loaded to the stress level corresponding to point B, it returns to point B exactly, forming a closed hysteretic loop BCB. The shape of the hysteretic loop is determined by the viscosity and plasticity of the rock. Although the loading and unloading paths are different for viscoelastic deformation, there is no plastic deformation, and the hysteretic loop is closed. The rock behaves as a viscoelastic material; it may be assumed that the rock is viscoelastic at this stage.

Thus, in the process of unloading from point B to point C and then loading back to point B, the deformation occurs as viscoelastic deformation without loss of elastic energy, and the energy loss during this period is the work done by the damping force [40,41]. It is assumed that there is no heat exchange between the specimen and the outside during the test, and energy such as thermal radiation and acoustic emission is ignored. The area of hysteresis loop BCB is the strain energy dissipated by overcoming the rock viscosity (including liquid viscosity and interface friction), namely damping energy.

(7) $$U_{dzi}=\int_{\varepsilon_{C}}^{\varepsilon_{B}}(\sigma_{i+1}^{+}-\sigma_{i}^{-})d\varepsilon_{i}$$

The dissipated energy minus the damping energy is the damage energy causing rock damage:

(8) $$U_{dsi}=U_{di}-U_{dzi}=\int_{\varepsilon_{O}}^{\varepsilon_{A}}\left(\sigma_{i}^{+}-\sigma_{i}^{-}\right)d\varepsilon_{i}-\int_{\varepsilon_{C}}^{\varepsilon_{B}}(\sigma_{i+1}^{+}-\sigma_{i}^{-})d\varepsilon_{i}$$

where $U_{dsi},U_{dzi}$ are the damage energy and damping energy of the *i*th cycle, respectively.

### 4.3. Analysis of Energy Evolution under Cyclic Loading

To study the relationships between input energy, elastic energy, and dissipated energy of rock under cyclic loading, the evolution laws of input energy, elastic energy, dissipated energy, energy storage ratio, and energy dissipation ratio were obtained using the theoretical method described in Section 4.1 and Equations (3)–(6) and (9).

The energy dissipation ratio and energy storage ratio are expressed as

(9) $$\eta_{1}=\frac{U_{ei}}{U_{i}},\eta_{2}=\frac{U_{di}}{U_{i}},\eta_{1}+\eta_{2}=1$$

where $\eta_{1},\eta_{2}$ are the energy storage ratio and energy dissipation ratio, respectively.

**Figure 7:**
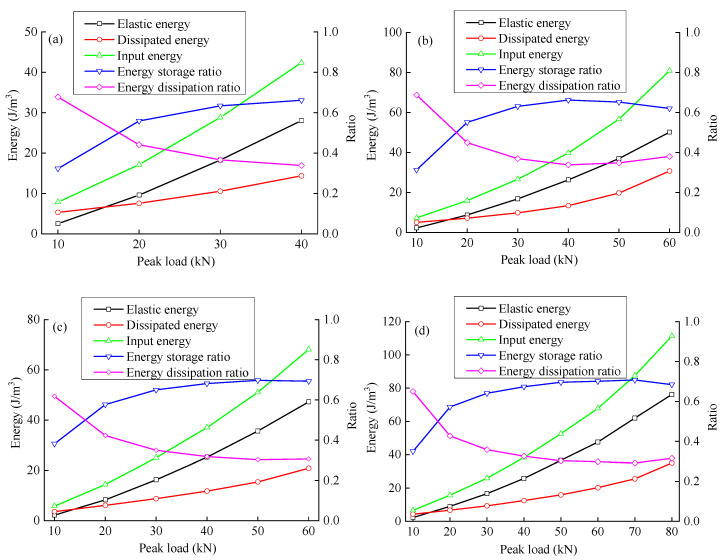

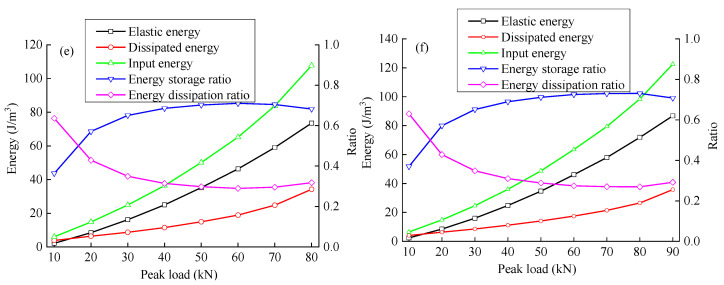
Evolution curves of input energy, elastic energy, dissipated energy, energy storage ratio, and energy dissipation ratio. ((**a**–**f**): X-0°, X-30°, X-45°, X-60°, X-90°, and X-intact, respectively).

Figure 7 shows the energy distribution of the sample; the input energy, elastic energy, and dissipated energy of the sample increase with an increase in peak load. When the peak load is large, the input and dissipated energy growth rates increase noticeably. The energy storage ratio exhibits a trend of first increasing and then decreasing; the energy dissipation ratio exhibits an opposite trend because the elastic deformation capacity of the rock gradually increases as the cracks in the primary pores are compressed by the load. Sandstone mainly exhibits elastic deformation, and more energy is stored in the form of elastic energy. The energy storage ratio increases with an increase in the load to a certain level, while the propagation speed of the new crack is accelerated, the stiffness of the rock decreases, the elastic deformation ability decreases, and the energy storage capacity decreases, increasing the energy dissipation ratio.

To study the relationships between dissipated energy, damage energy, and damping energy of rock under cyclic loading, the evolution laws of damping energy, damage energy, damping energy ratio, and damage energy ratio are obtained using the theoretical methods in Section 4.2 and Equations (7), (8) and (10).

The damping energy ratio and damage energy ratio are expressed as

(10) $$\eta_{3}=\frac{U_{dzi}}{U_{di}},\eta_{4}=\frac{U_{dsi}}{U_{di}},\eta_{3}+\eta_{4}=1$$

where $\eta_{3},\eta_{4}$ are the damping energy ratio and damage energy ratio, respectively.

**Figure 8:**
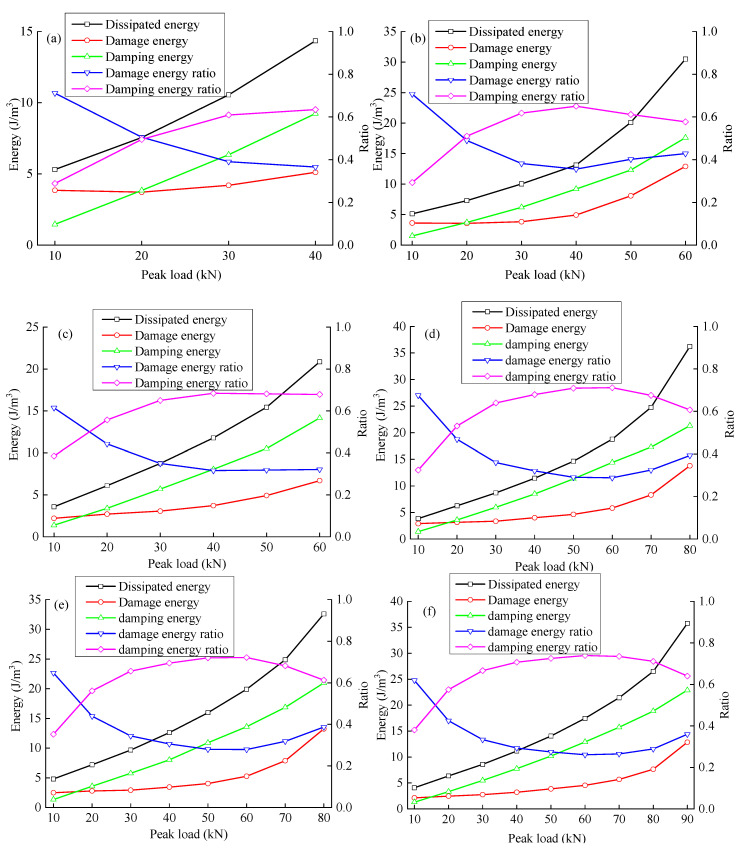
Evolution curves of dissipated energy, damping energy, damage energy, damping energy ratio, and damage energy ratio ((**a**–**f**): X-0°, X-30°, X-45°, X-60°, X-90°, and X-intact, respectively).

The damping energy generally shows an approximately linear trend with an increase in the peak load. Figure 8 shows that the damping energy of sandstone is affected mainly by the load. The analysis shows that the viscosity of rock comes mainly from the viscosity of liquid and the friction between cracks and pores (this is dry rock, so liquid viscosity can be ignored). The interface friction effect has a linear relationship with the load; the damping energy is generally approximately proportional to the peak load.

The damage energy increases slowly to a certain value with the peak load and then increases quickly, indicating that rock damage occurs at each stage of deformation failure. An internal crack develops slowly before reaching the mutation point of damage energy. The damage energy of rock increases slowly; after reaching the mutation point, the crack expands and connects, and the damage energy increases rapidly.

The damping energy ratio first increases and then decreases with an increase in peak load; the damage energy ratio first decreases and then increases with an increase in peak load. With an increase in the peak load, the microcrack pores in the rock initiate and expand, the friction between interfaces increases, and the damping energy of the rock increases faster than the damage energy. When the peak load approaches the failure strength, the cracks connect rapidly. Although the interface and damping energy increase, more energy is used to form the fracture surface, and the increase in damage energy is faster than the increase in damping energy.

Except for the first cycle, most of the dissipative energy in each cycle is dissipated in the form of work overcoming viscosity. With an increase in load, the proportion of damage energy increases noticeably, indicating that the load level accelerates the growth of new cracks and the formation of the fracture surface, leading to an accelerating rate of damage accumulation.

### 4.4. Energy Evolution Law under the Influence of the Crack Angle

**Figure 9:**
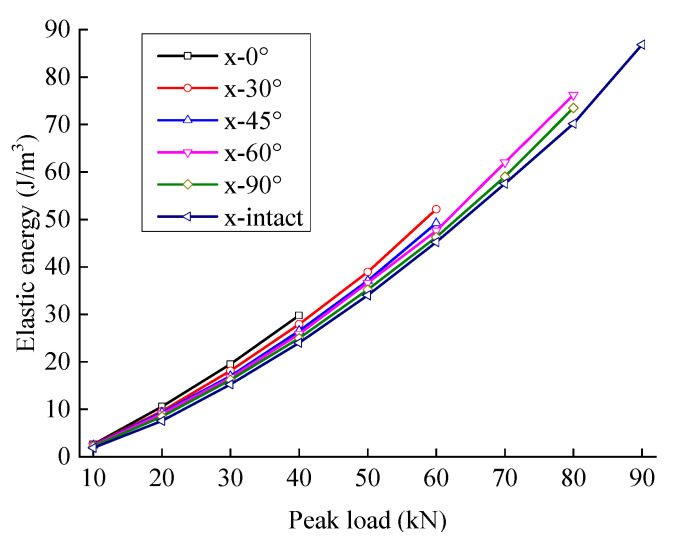
Elastic energy evolution curve of sandstone with different crack angles.

**Figure 10:**
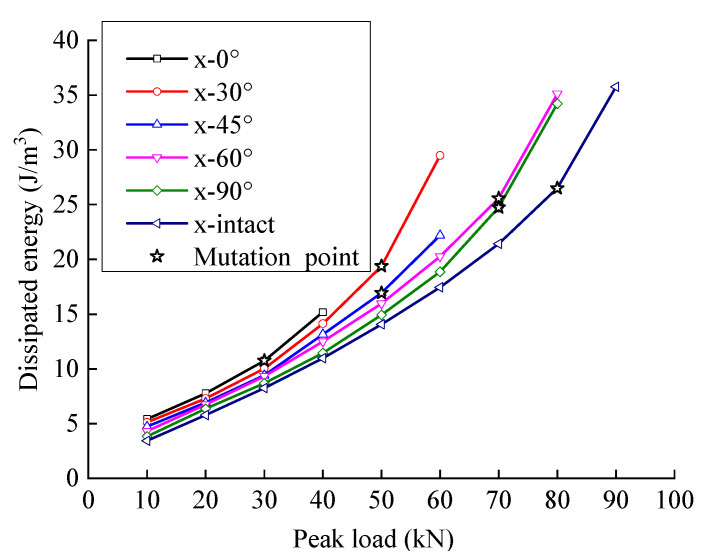
Dissipated energy evolution curve of fractured sandstone with different crack angles.

The energy evolution of sandstone samples is different with different rock crack angles. Figure 9 and Figure 10 show the variation laws of elastic energy and dissipated energy for fractured sandstone with different angles with a change in peak load. With different crack angles, when the peak load of sandstone is low, the difference in elastic energy is not great. With an increase in peak load, each specimen’s elastic energy gap gradually widens. A smaller crack angle produces a higher elastic energy curve for fractured sandstone; that is to say, with the same peak load level, the elastic energy increases with a decrease in the crack angle.

The dissipated energy increases stably at first and then rapidly with the peak load, indicating that the dissipated energy increases sharply when the peak load reaches a certain level (the slope mutation points are indicated in Figure 10). With the same peak load, the dissipated energy increases with an increase in the crack angle.

**Figure 11:**
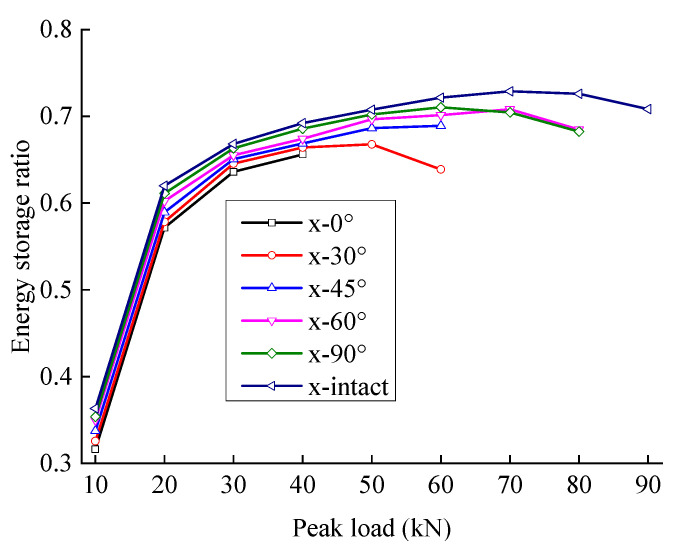
Energy storage ratio evolution curve of fractured sandstone with different crack angles.

**Figure 12:**
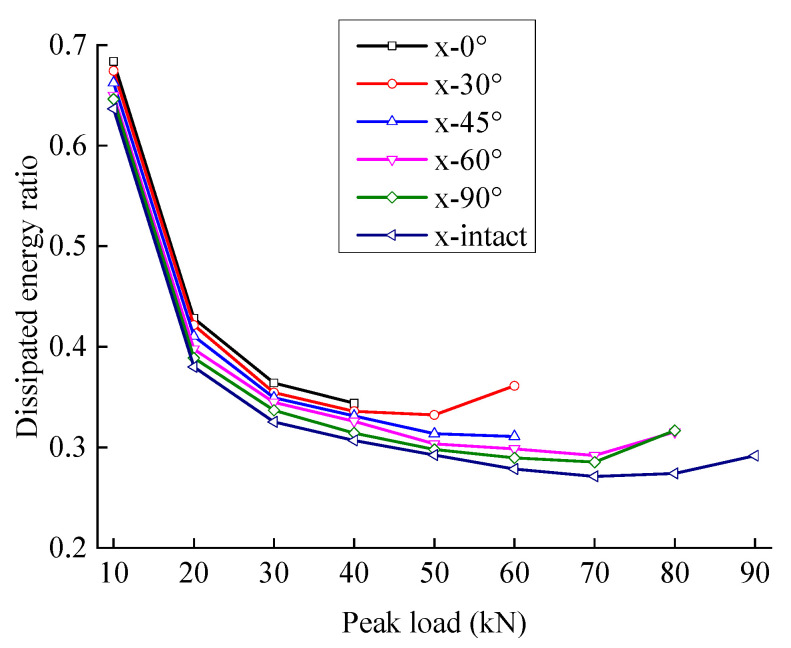
Energy dissipation ratio evolution curve of sandstone with different crack angles.

Figure 11 and Figure 12 show the relationship between energy storage ratio, energy dissipation ratio, and the peak load of fractured sandstone with different crack angles. With the same peak load, the energy storage ratio of sandstone increases with an increase in crack angle, and the energy dissipation ratio decreases (the energy storage ratio of the complete specimen is the largest, and the energy dissipation ratio is the smallest), indicating that the cracks weaken the elastic energy storage capacity and enhance the energy consumption capacity. Moreover, the smaller the crack angle is, the more severe the weakening of the energy storage capacity and the larger the energy dissipation ratio.

**Figure 13:**
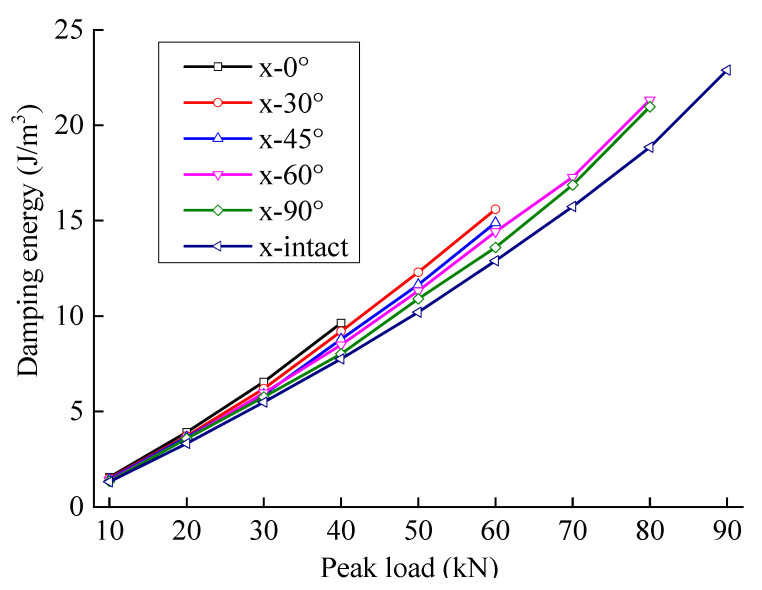
Damping energy evolution curve of sandstone with different crack angles.

**Figure 14:**
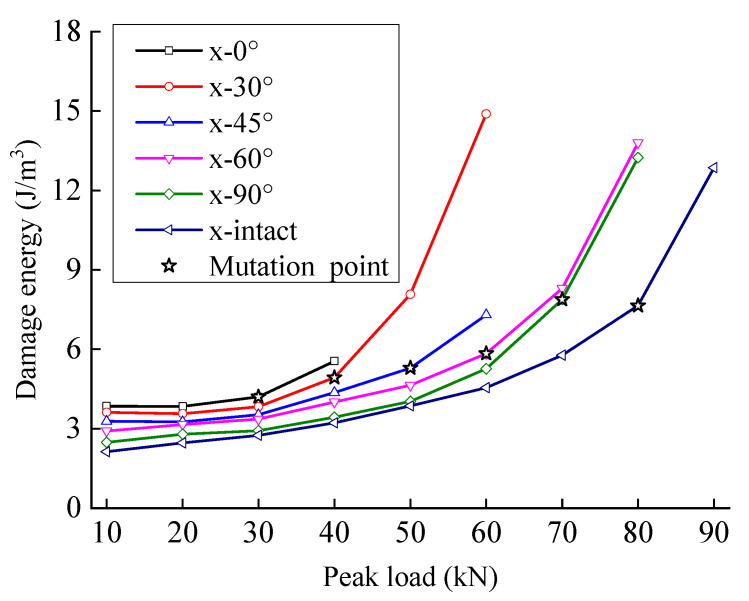
Damage energy evolution curve of sandstone with different crack angles.

Based on the division theory of dissipated energy, it is found that not all dissipated energy directly causes damage, but a large part of the energy is used to overcome the viscosity of the rock. Figure 13 and Figure 14 show the variation law of the fractured sandstone’s damping energy and damage energy with different crack angles with a change in peak load.

In Figure 13, with an increase in peak load, the damping energy gap of fractured sandstone gradually widens with different angles. The smaller the angle is, the higher the damping energy curve of fractured sandstone. A smaller crack angle produces greater damping energy with the same peak load.

In Figure 14, with the same peak load, a larger fracture angle produces smaller damage energy. The damage energy of fractured sandstone with different angles increases approximately synchronously and grows slowly when the peak load is small. When the peak load reaches a certain level, the damage energy increases rapidly, and the mutation point of the damage energy is approximately 4–6 J/m^3^. There is generally a threshold for damage accumulation, beyond which damage accumulates faster. This shows that with the increase of damage energy, the damage accumulation of rock gradually approaches failure. When the stress increases to a certain extent, the damage energy increases rapidly, indicating that the failure process of rock is accelerated. The existence of cracks is equivalent to initial damage; smaller angles produce greater initial damage. With an increase in crack angle, the mutation point of damage energy in fractured sandstone increases. Damage energy is the internal cause of rock damage, by analyzing the evolution law of damage energy, can more clearly judge the damage evolution of rock, and provide a reference for predicting rock damage and failure through damage energy in the later stage.

**Figure 15:**
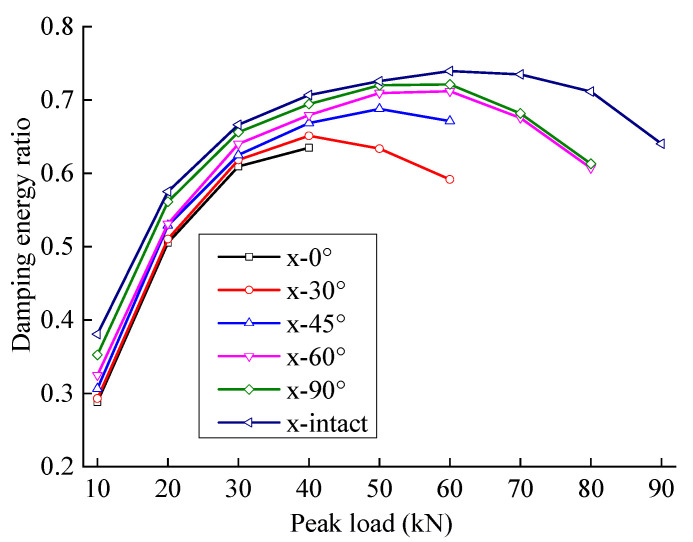
Damping energy ratio evolution curve of sandstone with different crack angles.

**Figure 16:**
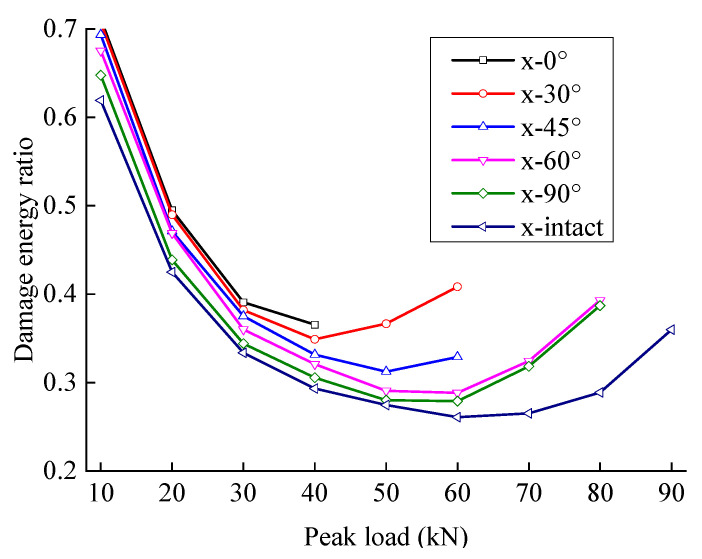
Damage energy ratio evolution curve of sandstone with different crack angles.

Figure 15 and Figure 16 show the variation laws of the damping and damage energy ratios for fractured sandstone with a change in peak load. The damage energy ratio decreases, and the damping energy ratio increases with an increase in crack angle. With a larger crack angle, more dissipated energy is used to overcome the sandstone viscosity, less dissipated energy is used to cause rock damage, and the energy index is closer to that of the complete specimen. The analysis shows that fractures reduce the effective bearing surface, increasing the effective stress inside the sandstone, which leads to an increase in internal friction and the failure of the structural plane. Thus, a smaller crack angle results in a larger energy index with the same peak load.

The damping and damage energy ratios are sensitive to the fracture angle. When the fracture angle is less than 45°, it greatly influences the energy ratio. When the fracture angle exceeds 60°, it has little effect on the energy ratio, which is close to that of the complete specimen.

## 5. Conclusions

Laboratory tests were conducted on sandstone with different crack angles to study the mechanical properties and fracture mode under cyclic loading. In this study, the energy storage ratio, energy dissipation ratio, damping energy ratio, damage energy ratio, and other energy parameters were defined. The energy evolution law of fractured sandstone considering rock viscoelastic deformation was also analyzed. The major conclusions of this study are presented as follows.

1. Under cyclic loading, the deformation modulus of fractured sandstone first increases and then decreases with the peak load. The lateral expansion coefficient is generally positively correlated with the peak load; with an increase in the fracture angle, the deformation modulus increases, and the lateral expansion coefficient decreases.
2. The energy dissipated by rock viscoelasticity was considered under cyclic loading and unloading. The dissipated energy was divided into the damping energy consumed to overcome rock viscoelasticity and damage energy causing damage by damage by viscoelastic deformation theory. An energy analysis model considering damping energy and damage energy is established.
3. The elastic energy increases approximately linearly with the peak load, and the energy storage ratio first increases and then decreases with an increase in the peak load. A smaller crack angle produces greater elastic energy and a smaller energy storage ratio with the same peak load. The dissipated energy increases slowly at first and then rapidly with the peak load; the energy dissipation ratio first decreases and then increases with an increase in peak load. With the same peak load, a smaller crack angle produces greater dissipated energy and a greater energy dissipation ratio.
4. The damping energy increases approximately linearly with the peak load; the damping energy ratio first increases and then decreases with an increase in peak load. A smaller fracture angle produces greater damping energy and a smaller damping ratio with the same peak load. The damage energy increases slowly at first and then rapidly with the peak load; the damage energy ratio first decreases and then increases with an increase in peak load. With the same peak load, a smaller fracture angle produces greater damage energy and a greater damage energy ratio.

## Figures and Tables

**Table 1:** Specimen styles and test results for group X.

Specimen	Crack Angle/°	Crack Length/mm	Horizontal Projection Area/mm^2^	Peak Stress/MPa	Peak Strain	Number of Cycles	Uniaxial Compressive Strength/MPa
X-0°	0°	16	786.2	24.7	0.00875	4	27.5
X-30°	30°	16	683.8	30.5	0.0088	6	32.3
X-45°	45°	16	560.9	35.6	0.00803	6	36.3
X-60°	60°	16	398.3	40.75	0.00857	8	42.4
X-90°	90°	16	50	44.3	0.00956	8	47.5
X-Intact	/	16	0	47.4	0.00971	9	50.8

## Data Availability

Not applicable.
